# Extramammary Paget Disease: a Therapeutic Challenge, for a Rare Entity

**DOI:** 10.1007/s11912-023-01434-0

**Published:** 2023-07-08

**Authors:** Jesús Chamorro Pérez, Alfonso Cortes Salgado, Belén Pérez-Mies, Jose Antonio Domínguez Rullán, Odile Ajuria-Illarramendi, Eva María Guerra Alia, Juan José Serrano Domingo

**Affiliations:** 1 Medical Oncology Department, Ramón y Cajal University Hospital, Carretera de Colmenar Viejo, Km 9.100, 28034 Madrid, CP Spain; 2 Pathology Department, Ramón y Cajal University Hospital, Carretera de Colmenar Viejo, Km 9.100, 28034 Madrid, CP Spain; 3 Radiation Oncology Department, Ramón y Cajal University Hospital, Carretera de Colmenar Viejo, Km 9.100, 28034 Madrid, CP Spain; 4 Nuclear Medicine Department, Ramón y Cajal University Hospital, Carretera de Colmenar Viejo, Km 9.100, 28034 Madrid, CP Spain

**Keywords:** Extramammary Paget disease, Imiquimod, Radiotherapy, Chemotherapy, HER-2

## Abstract

**Purpose of Review:**

Extramammary Paget disease (EMPD) is a rare entity which is more frequently localized at the vulva, though it only accounts for 1–2% of vulvar neoplasms. It is a primary cutaneous adenocarcinoma whose cell of origin is still a matter of controversy: it can either arise from apocrine/eccrine glands or from stem cells. The diagnosis demands a biopsy and entails a histopathological analysis by which cells show similar characteristics as breast Paget disease.

**Recent Findings:**

Treatment approach can entail surgery, radiotherapy, photodynamic therapy, systemic chemotherapy, and topical chemotherapy. For metastatic disease, many different chemotherapy regimens have been explored and even targeted therapy can play an important role in this disease. Since almost 30–40% of patients overexpress HER-2, trastuzumab and anti-HER-2 therapies can be employed in this setting.

**Summary:**

Due to its low incidence, there is almost no specific evidence on therapeutic interventions for this disease. Thus, there is a neat unmet need for molecular characterization of EMPD and diagnostic tools that allow clinicians to guide treatment both in the early and in the advanced disease settings. In this review, we aim to summarize available evidence about diagnosis and treatment of EMPD, both localized and metastatic, and to provide a comprehensive analysis that may help clinicians for therapeutic decisions.

## Introduction

Vulvar cancer is the 4th gynecological neoplasm by frequency in women in the USA after uterus, ovary, and cervix. However, it is a very rare pathology, with about 6000 cases per year and a mortality of about 1000 cases in the USA [1]. Squamous cell is the most frequent histology, representing up to 75% [2]. Other histologies include melanoma, basal cell carcinoma, adenocarcinoma of the Bartholin glands, sarcomas, and Paget’s disease.

Paget’s disease was described by Sir James Paget in 1874 [3]. The first case of extramammary Paget’s disease (EMPD) was described by Crocker in 1889 [4] being the vulva the most affected region (54–65%) [5, 6]. In contrast, EMPD accounts for only 1–2% of vulvar neoplasms [7, 8]. Although it may be asymptomatic, the majority present with pruritus, burning, or decreased sensitivity [9••], being white postmenopausal women the most frequently population affected [10]. In general, it is well delimited, with slightly raised edges and a red background, often with small pale islands. It is usually multifocal and can appear anywhere on the vulva, perineum, or inner thighs.

The diagnosis is histological, and there may be foci of invasive adenocarcinoma in up to 4–17%, inside and/or below the lesion [11, 12]. In addition, it can be associated with other malignancies such as breast, rectum, bladder, urethra, cervix, or ovary in up to 10–42% of cases [13, 14].

## Histopathology and Immunohistochemistry

**Fig. 1:**
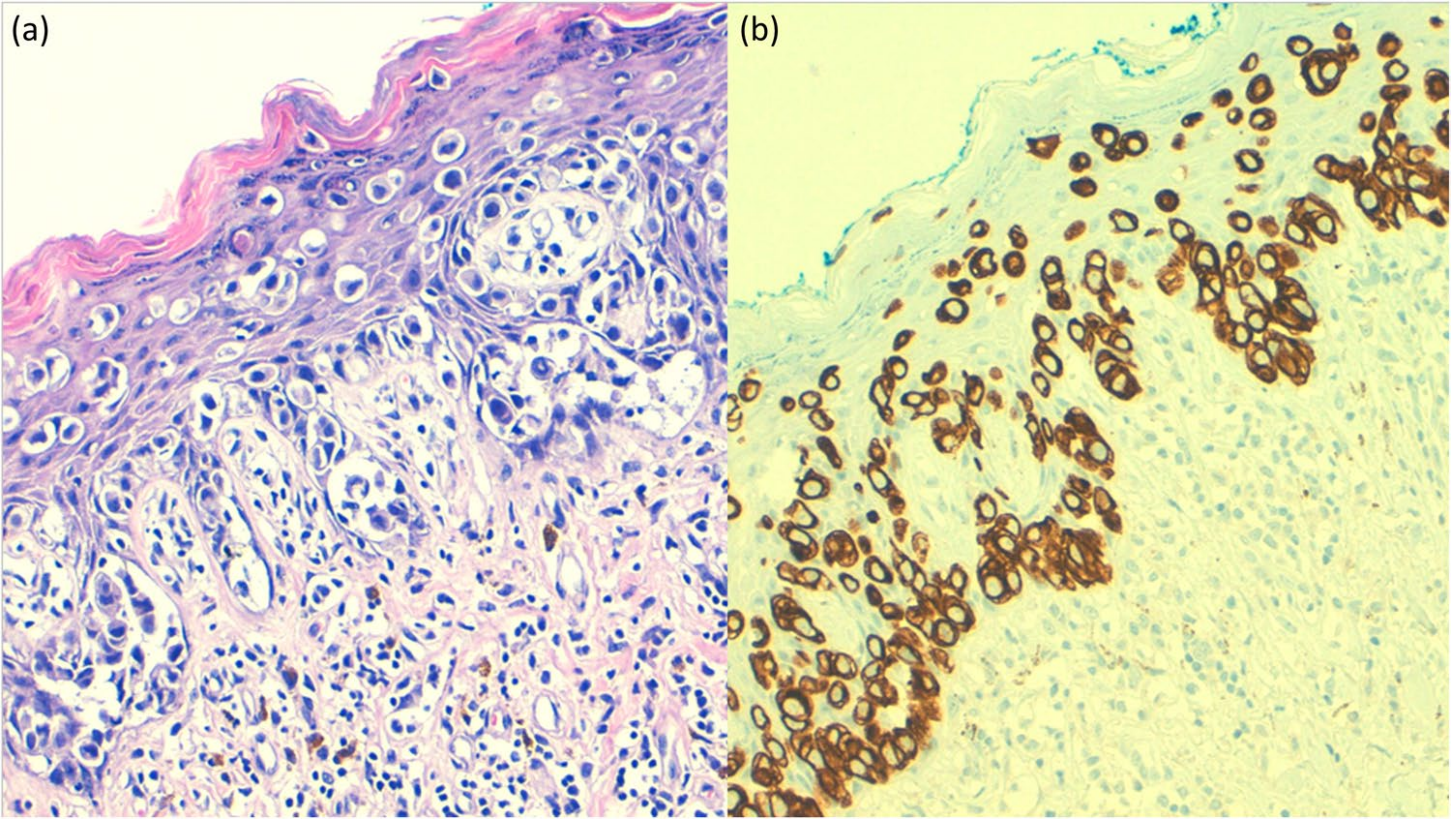
**a** Paget disease (hematoxylin and eosin): nests of pale-staining tumor cells are arranged singly or in small cluster along the vulvar epidermis. **b** CK7 immunohistochemistry in a Paget disease

The diagnosis is histopathological. The tumor cells of EMPD, as well called Paget’s cells, have abundant pale cytoplasm and large nuclei with a prominent, vesicular nucleus, with unusual mitotic figures [15] (Fig. 1a). The tumor usually locates in the epidermis, with occasional dermal invasion. This entity can be confused with other neoplasms, such as melanoma, Bowen’s disease, or sebaceous carcinoma, which can show similar morphological findings. In these cases, periodic acid-Schiff (PAS) and Alcian blue stains can help to make an accurate diagnosis, due to the abundant mucin production of this entity. Immunohistochemistry may provide helpful information to make a proper diagnosis. On the one hand, EMPD is usually positive for carcinoembryonic antigen (CEA), cytokeratin (CK) 7 (Fig. 1b), CAM5.2, and gross cystic disease fluid protein (GCDFP15). On the other hand, it is negative for S100 protein and other melanocytic markers (melan-A, MITF, HMB45, etc.) [16].

Notwithstanding, primary and secondary EMPD can show different immunophenotypes [17–19]. Usually, primary EMPD is positive for CK7 and GCDFP15 (with CK20 negative). Nevertheless, some locations may present different immunohistochemistry profiles. For instance, there are cases of primary perianal EMPD with GCDFP15-positive and CK20-negative [17], whereas other cases of primary vulvar EMPD show both markers positive [19].

A recent study has described a high presence of EpCAM (epithelial cell adhesion molecule, CD326) in EMPD and a negative correlation between its expression levels and the presence of distant metastasis [20].

It has also been reported a frequent overexpression of cyclin-dependent kinase 4 and cyclin D1 in EMPD [21], which could play a role in the disease treatment [22].

## Prognostic Factors

Several factors of poor prognosis have been identified in EMPD. Vaginal localization had higher mortality than those localized in vulva or lips, as well as those with metastatic involvement (HR 3.26, both). Male sex (HR 1.42), being older (HR 1.09), and different races from white or black (HR 0.81 Vs. 0.65) also have worse prognosis [23].

In another analysis, receiving radiotherapy was related with poorer prognosis (HR 1.6 vs. 1.09). Therefore, they re-analyzed the risk factors in a separate multivariate analysis according to whether they had received radiotherapy or not. The location in the vagina and the presence of metastases continued to be a factor of poor prognosis, while having undergone surgery was a factor of good prognosis in all patients. Moreover, the race ceased to be a prognostic factor and sex only continued to be so in patients who did not receive radiotherapy [6].

Other authors had previously described tumor depth, lymphovascular invasion, and the presence of metastases as factors of poor prognosis [24, 25], likewise the number of affected lymph nodes [24, 26•], or positive sentinel lymph node biopsy (SLNB) [26•].

## Positron Emission Tomography (PET)–Computed Tomography (CT)

There is limited evidence about the role of PET-CT in EMPD to assess the presence of disease at a distance [27,28,29,30,31,32, 33•].

A retrospective study reviewed 10 cases with newly diagnosed disease or suspected recurrence [32], with two cases of recurrence confirmed and one false positive. Nodal involvement was detected in 6 cases, with 20.4% of them measuring less than 1 cm. Other metastatic locations included bone, liver, lung, and adrenal glands. Compared to the conventional study, 4 out of 10 patients experienced changes in staging and the treatment was modified based on the findings in 3 of them [32].

Inversely, other study analyzed the relationship of SUVmax with the presence of lymph node metastases. They evaluated 15 cases of patients with localized EMPD in which pre-surgical PET and SLNB had been performed. The SLNB was positive in 7 patients (of 37 nodes analyzed). These positive nodes showed a SUVmax between 3.7 and 11.7, while it was ≤1.74 in the negative nodes. Three nodes were suspected in CT scan, resulting in a negative histological study, while 2 nodes with no suspicion of malignancy in the PET scan were affected. The authors proposed a SUVmax cut-off point of 2.5 [33•].

According to the groups established in Table 1, there were no cases of false positives or false negatives. Based on their results, the authors concluded that PET is a valid tool for the detection of regional lymph node involvement, suggesting the need for a SLNB in cases with a SUVmax ≤2.5.

**Table 1:** Groups established by Fujiwara et al.

		Histology	
		**+**	**-**
PET-CT	**+**	7	0
	**-**	0	19

*PET-CT*, positron emission tomography-computerized tomography

Although there is no robust evidence to recommend standard use of PET-CT for the evaluation of both localized and metastatic EMPD (mEMPD), it seems to provide useful information for disease staging.

## Sentinel Lymph Node Biopsy

The need to carry out SLNB is controversial and the available evidence shows dissimilar results. Hatta et al. published a case series of 13 patients who underwent SLNB (unilaterally or bilaterally), being positive 4 of them. Three of the four patients with a positive SLNB had invasive disease, while all patients with in situ disease had a negative SLNB. Those 3 patients underwent lymphadenectomy, being positive all of them. Subsequently, two of them presented distant metastases [34].

Ogata presented similar results in a case series of 59 patients in whom surgery and SLNB were performed. The SLNB was positive in 10 patients, performing a subsequent lymphadenectomy in 9 of them (the other patient refused it). There was a total of 27 nodes out of 139 with a median of affected nodes per patient of 2.4. The probability of a positive SLNB was correlated with the level of invasion of the primary tumor. The 5-year overall survival (OS) in patients with a positive SLNB was 24%, while in the negative SLNB group was 100%. Similarly, the number of affected nodes was also found as a prognostic factor, with a 5-year survival rate of 100% for those who had 2 or less and 0% for those who had 3 or more [26•].

However, Fujisawa et al. presented divergent results. They analyzed a total of 151 patients who underwent SLNB, performing lymphadenectomy in 113 of them. Of the 107 patients without clinical lymphadenopathy, 16 had lymph node involvement. Although the level of invasion was again shown as a poor prognosis factor, there were no differences between positive or negative SLNB. The relapse rate was also similar among patients with positive and negative SLNB [35].

Despite discordant results from the Fujisawa study, it seems that the level of invasion and having affected nodes (either after lymphadenectomy or SLNB) worsen the prognosis. Nevertheless, performing a lymphadenectomy after a positive SLNB has not clearly been demonstrated to improve these patients’ prognosis. Given the comorbidities associated with lymphadenectomies, we believe that each case must be individualized when indicating a SLNB.

Other authors have suggested the use of ultrasound imaging to identify suspicious lymph nodes, based on morphology and perfusion criteria (balloon shape, loss of central echo, and presence of peripheral perfusion) which are as useful as the longitudinal/transversal ratio of 2 or less. They also propose to perform a lymph node biopsy instead of immediate lymphadenectomy [36].

## Surgery

Lesion excision is the standard treatment for localized disease, avoiding radical vulvectomy, but ensuring margins of 2 cm. The degree of depth of the lesion and lymphovascular involvement are poor prognosis factors [15], so some authors have proposed lymphadenectomy if there are foci of adenocarcinoma [37].

Sarmiento et al. evaluated the need for lymphadenectomy in a study where 34 patients were classified into 4 groups: carcinoma in situ (CIS), microinvasion to the papillary dermis (MIPD), invasion of the reticular dermis (IRD), and invasion of subcutaneous tissue (IST) [38]. Patients with CIS or MIPD did not undergo lymphadenectomy and no disease-related deaths were reported. Among the 6 patients with IRD, 4 underwent lymphadenectomy, with 3 of them presenting node metastases, who would later die. All the 6 patients with IST underwent lymphadenectomy, and all of them had node involvement, dying due to the disease. Based on this, the authors recommended lymphadenectomy if the lymph node biopsy is positive and in case of IRD or IST, being unnecessary in cases of CIS or MIPD. Notwithstanding, no survival improvement was demonstrated with lymphadenectomy [38]; thus, its execution should be individualized.

Despite performing surgery with free margins, up to 12–60% of cases can present local relapse [12, 39–45]. In these cases, a new resection can be attempted. Since it is possible to need 5-cm margins of “apparently normal skin” to achieve free margins, some authors have proposed resection by Mohs surgery to achieve it [46] with good results in retrospective series [47]. A recent study reported 11% of recurrences in 19 patients treated with Mohs surgery compared with 36% of recurrences in 45 patients treated with conventional surgery [48••].

## Non-surgical Treatments

In those cases, in which surgical resection is not possible, due to patient comorbidities or unresectable disease, there are alternatives such as radiotherapy, photodynamic therapy, systemic chemotherapy, and topical chemotherapy, especially imiquimod.

Imiquimod binds to the Toll-like receptor 7, inducing an innate and cell-mediated immune response [49]. There are several articles reporting benefits in the use of 5% topical imiquimod. Overall response rates (ORR) between 56 and 100% have been described, with a recurrence rate up to 62% at the end of treatment, although most of the lesions responded again after reintroducing imiquimod [49–57]. Three times a week application was the most recurrent schedule in those studies. The most frequent toxicities are erythema, erosions, and local pain. Some patients may require the use of concomitant topical corticosteroids or anesthetics due to these adverse events.

There is a recently published study which explored the application of 5% imiquimod in patients with non-invasive recurrent vulvar EPMD (vEMPD). In this multicenter, prospective, observational, and open-label study, 24 patients were included, receiving imiquimod 3 times a week for 16 weeks. The reported results showed an ORR of 82.6%, with 10 histological complete responses (CR) and 7 partial responses [58••].

Photodynamic therapy is a non-invasive treatment that uses photosensitizing drugs such as 5-aminolevulinic acid or 16% methyl-aminolevulinic acid. The area where it is applied is afterwards exposed to light with the appropriate wavelength that destroy tumor cells. The ORR rages between 53 and 68% but there is a high recurrence rate, between 56 and 100% [59–61]. The predominant side effects include burns and pain.

### Radiotherapy

Tolia et al. published in 2016 a deep review about the use of radiotherapy in vEMPD [62]. They reviewed 10 articles between 1992 and 2015 with a total of 57 patients with invasive vEMPD treated with radiotherapy. The authors concluded that surgical resection continues to be the standard of treatment in vEMPD, although recurrence rates after it varies from 12 to 60% [12, 31–37].

Hence, they recommend the use of radiotherapy with adjuvant intention in case of affected margins, locoregional lymph node involvement, multifocal disease, or presence of adenocarcinoma. Likewise, they also consider the use of radiotherapy as an alternative in cases where surgery is not possible. Given the heterogeneity of the reported cases, they do not establish an optimal dose of radiotherapy, but for definitive treatment, it ranges between 44 and 81.6 Gy in the primary tumor and between 44 and 70.2 Gy in the locoregional lymph nodes, and in the adjuvant setting, it ranges between 44 and 64.8 Gy and 32 and 50.4 Gy, respectively [62].

There is a more recent systematic review, in which 195 patients were analyzed [63••]. For definitive treatment, doses of radiotherapy between 30 and 80.2 Gy (3–43 fractions) were observed, with CR rates of 50–100% and relapse/persistence rates between 0 and 80%. The authors recommend, at least, doses of 60 Gy. In the adjuvant setting, the doses used were between 32 and 64.8 Gy (20–30 fractions) with relapse rates of 0–62.5%. The authors recommend doses between 45 and 60 Gy in cases of dermal invasion by the tumor of proximal involvement to the margins, and at least 60 Gy in cases of positive postoperative margins or nodal involvement. This study has the same limitations as that of Tolia et al. with respect to the great heterogeneity it presents regarding tumor staging and the different techniques used, as well as the volumes and doses of radiotherapy used.

In a large retrospective series that collected the data from Surveillance, Epidemiology, and End Results (SEER) program of 1439 patients with EMPD, the vulva was the most commonly affected region (781 cases, 81.3%) [6]. In this series, 1230 patients (86.4%) underwent surgery. The majority of patients (1335, 93.6%) did not receive radiotherapy. Of those who underwent radiotherapy (92, 6.4%), 51 received it as adjuvant treatment after surgery and 40 as sole treatment. Patients who received radiotherapy had a disease-specific survival (DSS) of 134.7 months (95% confidence interval (CI) 113.7–155.6), being lower than those who did not receive radiotherapy (342.1 months 95% 330.5–353.7, *p*<0.001). Patients who underwent surgery exclusively showed better prognosis with a DSS of 346.8 months (95% CI 335–358.6) compared to patients who did not undergo surgery or radiotherapy (median DSS 255.1 months, 95% CI 221.1–289.2, *p*=0.002), to patients who only received radiotherapy (median DSS 143.3 months, 95% CI 119.2–167.5, *p*=0.004), and to patients who underwent surgery and radiotherapy (median DSS 120.6 months, 95% CI 93.6–147.6, *p*<0.001).

This detriment in DSS in those patients who received radiotherapy (including those who also underwent surgery) could not be explained by the authors, in spite of adjusting for sex, age, location, and stage of the disease. The authors suggested radiotherapy may induce biological alterations in the tumor that render it greater aggressiveness, although they could not prove it. They also concluded that the heterogeneity of the data could affect the analysis and its interpretation.

By their hand, Tolia et al. explained that these results could be due to the fact that the use of radiotherapy was related to advanced stages or recurrent disease. They also believed that the interpretation of the data may be affected by the heterogeneity of radiotherapy schemes, dose, and fields of application [62].

By our hand, we agree with the conclusions of Tolia et al., reserving RT for those situations in which surgery is not possible, or as an adjuvant treatment in case of margins affected, locoregional lymph node involvement, multifocal disease, or presence of adenocarcinoma.

## Systemic Treatment for Advanced Disease

There is no standard treatment for mEMPD and no randomized clinical trials have been performed in order to settle the optimal treatment for this disease. Given its low frequency, the vast majority of the available evidence proceeds from multiple isolated cases and small case series.

The first report cases date from the late 1980s and early 1990s. Piedbois published a case of a woman who presented a CR after been treated with mitomycin-C, vincristine, 5-fluorouracil, doxorubicin, cisplatin, and bleomycin [64]. Balducci would only use mitomycin-C and 5-fluorouracil in a male patient, who obtained a CR as well [65]. In the early 1990s, the case of a woman treated with mitomycin-C, cisplatin, and vincristine would also be published, reaching a partial response (PR) for 5 months [66]. In the frame of this thinking, the FECOM scheme was established, consisting in a combination of mitomycin-C, epirubicin, vincristine, carboplatin, and 5-fluorouracil. It was firstly used in a case with a PR and a 15-month survival [67]. Additionally, in a series of 7 patients, an ORR of 100% and a 1-year survival rate of 43% were reported with the use of FECOM schedule [68]. However, there are also reported cases of poor response [69, 70].

Combinations with fluoropyrimidines and platins have been widely explored in the last decade. Holger presented a case of a patient treated with 5-fluorouracil in combination with carboplatin with a CR for 12 months [71]. Subsequently, carboplatin was substituted in favor of cisplatin. Kariya et al. achieved a PR maintained for 16 months [72]. In a retrospective analysis of 22 cases, an ORR of 59% and a 1-year survival rate of 50% were obtained [73]. Kato et al. would also perform a retrospective analysis in 8 patients treated with this scheme, with an ORR of 50% and an OS of 19 months [74].

In 2019, a case of mEMPD treated with a non-previously described combination of topic 5-fluorouracil and systemic pemetrexed was reported. The disease showed a PR for more than 6 months [75].

Some cases treated with anthracyclines either in monotherapy or in combination have likewise been reported. A patient with cerebral involvement that did not respond to polychemotherapy and other therapies (tamoxifen, thalidomide) obtained a PR with liposomal doxorubicin and progression free survival (PFS) of 1 year [76]. Anecdotally, a case of complete remission of localized vEMPD in a patient who was simultaneously treated with anthracyclines and taxanes for a breast cancer has been reported [77].

However, at the beginning of the twenty-first century, taxanes became the most used drugs in mEMPD, frequently combined with 5-fluorouracil and carboplatin.

Docetaxel monotherapy has been broadly used as first-line treatment. Fujisawa would use a 2:1 week scheme with a PR maintained for 13 months [78], while other authors would use a 3:1 week scheme, obtaining in this case a CR after 9 cycles and PFS of 2 years [79]. Yoshino et al. would present a series of 13 patients using monthly docetaxel with an ORR of 58% (all of them were PR), a PFS of 7.1 months, and OS of 16.6 months, with a 1-year survival of 75% [80]. Docetaxel has been used likewise as second line and beyond. Oashi et al. published a series of 3 patients who received such therapy after progression to the FECOM scheme [68]. Other authors used it as a third-line treatment after progression to FECOM scheme and 5-fluorouracil plus cisplatin, achieving a PR maintained for 12 months [70].

Taxanes have also been combined with 5-fluorouracil, S-1 (consisting of tegafur, 5-chloro-2,4-dihydroxypyridine, and potassium oxalate), and platins.

Zhu et al. presented a series of 10 patients, of which 2 were treated with docetaxel alone, 2 in combination with cisplatin, and 3 in combination with 5-fluorouracil, achieving an ORR of 57% [29]. Because the data is not separately by schemes, it is difficult to draw conclusions from this study. A case of a patient treated with docetaxel and carboplatin reached a PFS of 13 months [81]. There is also larger series, with one patient treated with paclitaxel alone (PR) and 2 patients in combination with carboplatin (one progression and one stable disease) [82].

Some authors have used S-1 in combination with docetaxel. A series of 4 patients reported a PR for 30 weeks (after progression to FECOM) [69], a CR maintained for 1 year [83], and a CR and PR for more than 10 months [84]. The use of S-1 has been also used to reverse the resistance to docetaxel, achieving response in a total of 3 cases [85, 86]. Fukuda et al. also reported a series of 8 cases where patients were treated with docetaxel and S-1 after progression to docetaxel. They obtained 2 PR and a disease control rate (DCR) of 88%. Median PFS and OS were 8 and 10 months respectively [87].

Finally, Hirai et al. explored an unusual combination of cisplatin, epirubicin, and paclitaxel (PET regimen) in a series of 5 patients. Three patients received it as first-line regimen, one as second line and the other as third line. There were 4 PR and 1 PD, with a PFS of 8 months and an OS of 20.1 months. The dosing schedules were diverse: biweekly for one patient, 2 weeks on and 2 weeks off for two patients, and triweekly for another patient [88].

### Targeted Therapy

Overexpression of HER2 has been described in up to 30–40% of mEMPD. The first case was described in a breast Paget in 1989 [89], and 2 years later in EMPD [90]. It has been postulated that the alteration of this pathway could contribute to the progression of the disease, as all PI3K and ERK pathways [91].

We summarized in Table 2 the current evidence of HER2 overexpression prevalence in EMPD [88, 91,92,93,94,95,96,97,98,99,100,101,102,103••]. Among 486 cases of localized disease and 99 of metastatic disease, there is an overall expression of HER2 of 28.2% and 40% respectively.

**Table 2:** Prevalence of HER2 amplification in metastatic and localized extramammary Paget’s disease

First author	Year	No. of patients	Stage	HER2 positive	Detection methods
Takata [92]	1999	27	Localized	6 (22%)	IHC, FISH
		4	LNM	2 (50%)	
Tanskanen [93]	2003	21	Localized	11 (52%)	ICH, FISH
		2	LNM	1 (50%)	
Brummer [94]	2004	10	Localized	8 (80%)	IHC
Reich [95]	2005	6	Localized	4 (66.7%)	FISH
Ogawa [91]	2005	34	Localized	3 (8.8%)	IHC
Bianco [96]	2006	15	Localized	1 (6.67%%)	IHC, CISH
Plaza [97]	2009	47	Localized	15 (32%)	IHC
Richter [98]	2010	33	Localized	19 (57.6%)	IHC, FISH
Miyamoto [99]	2010	23	Localized	13 (69.5%)	IHC, FISH
		9	Metastatic	7 (77.7%)	
Hikita [100]	2012	17	Localized	4 (23.5%)	IHC, FISH
Tanaka [101]	2013	78	Localized	8 (10%)	IHC, FISH
		26	Metastatic	4 (15%)	
Kang [102]	2015	227	Localized	45 (18.3%)	IHC
		19	LNM		
Hirai [88]	2019	47	Metastatic	23 (49%)	IHC, FISH
Lu [103••]	2019	11	Metastatic	3 (27.2%)	FISH
Total		486	Localized	137 (28.2%)	
		99	Metastatic and LNM	40 (40%)	

*LNM*, lymph node metastases; *IHC*, immunohistochemistry; *FISH*, fluorescent in situ hybridization; *CISH*, chromogenic in situ hybridization

In the same way, we have summarized the efficacy of targeting treatment with anti-HER2 therapies in Table 3 [103••,^104^,^105^,^106^,^107^,^108^,^109^,^110^,^111^,^112^,^113^, ^114^•, 115]. Represent a total of 17 patients, with a marked response, including 4 cases of CR (20%) and 9 PR (45%). Most of patients were treated with an anti-HER2 therapy at first line (12, 60%) in combination with a taxane (8, 66.7%), and a median PFS of 12 months. An unusual treatment with pyrotinib has been reported, with a PR but only 2 months of follow-up [116]. This case has not been included in Table 3.

**Table 3:** Efficacy of targeted therapy against HER2

First author	Year	Treatment modality	QT added	Effect	Line of treatment	PFS, months	OS, months
Karam [104]	2008	Trastuzumab (300 mg/m^2^)		PR	First line	12	NR
Takahagi [105]	2009	Trastuzumab 2 mg/kg qw (LD 4 mg/kg)	Paclitaxel (80 mg/m^2^ qw)^a^	PR	First line	6	15
Hanawa [106]	2011	Trastuzumab 2 mg/kg (LD 4 mg/kg), 6 weeks on/2 weeks off	Paclitaxel (80 mg/m^2^ qw)^b^	PR	First line	14	NR
Wakabayashi [107]	2012	Trastuzumab 6 mg/kg q3w (LD 8 mg/kg)		CR	First line	>13	NR
Yoshimura [108]	2013	Trastuzumab 2 mg/kg qw (LD 4 mg/kg)	Paclitaxel	NR	Second line	4	14
Barth [109]	2015	Trastuzumab 6 mg/kg q3w (LD 8 mg/kg)		CR	First line	>12	NR
Zhang [110]	2015	Trastuzumab 6 mg/kg q3w		PR	Second line	>11	NR
Shin [111]	2016	Lapatinib^c^	Capecitabine^d^	NR	Second line	NR	NR
		T-DM1^c^		CR	Third line	12	
Watanabe [112]	2016	Trastuzumab 6 mg/kg q3w (LD 8 mg/kg)^e^		PR	First line	5	>17
		Trastuzumab 6 mg/kg (LD 8 mg/kg) and pertuzumab 420 mg/m^2^ (LD 840 mg/m^2^) q3w^e^	Docetaxel (75 mg/m^2^ q3w)	PR	Second Line	12	
Ichiyama [113]	2017	Trastuzumab 2 mg/kg qw (LD 4 mg/kg)	Paclitaxel (80 mg/m^2^ qw)	PR	First line	>30	NR
Lu [103••]	2019	Trastuzumab 2 mg/kg qw	Paclitaxel (80 mg/m^2^) and cisplatin (30 mg/m^2^) qw	CR	First line	17	NR
		Trastuzumab 360 mg q3w^f^	Paclitaxel (80 mg/m^2^ qw)	SD	Second line	5	NR
		Lapatinib^f^	Capecitabine	SD	Third line	5	
Sekiguchi [114•]	2020	Trastuzumab 2 mg/kg qw	Paclitaxel 80 mg/m^2^ qw	PR	First line	12	30
		Trastuzumab 2 mg/kg qw	Paclitaxel 80 mg/m^2^ qw	PD	First line	2	6
		Trastuzumab 2 mg/kg qw	Paclitaxel 80 mg/m^2^ qw	PD	First line	4	13
		Trastuzumab 2 mg/kg qw	Paclitaxel 80 mg/m^2^ qw	PD	First line	3	20
Kimura [115]	2020	Trastuzumab (biosimilar) 2 mg/kg q2w		SD	Second line	>6	13

*QT*, chemotherapy; *PFS*, progression free survival; *OS*, overall survival; *LD*, loaded dose; qm, once a month; *qw*, once a week; *q3w*, once every 3 weeks; *NR*, not reported; *CR*, complete response; *PR*, partial response; *SD*, stable disease

^a^In the first cycle, chemotherapeutic agent was docetaxel. Paclitaxel was added because there was no response with docetaxel

^b^Paclitaxel was added in the second cycle, because there was no response

^c^These regimens belong to the same patient

^d^Added when lapatinib dose was reduced because hepatic toxicity

^e^These regimens belong to the same patient

^f^These regimens belong to the same patient

Even though the evidence of anti-HER2 efficacy in this entity is not robust, due to its low incidence, determination of HER2 expression should be performed in all cases of mEMPD.

Overexpression of androgen receptors and estrogen have been described in 53.6–100% and 0–19.44%, respectively, being able to coexist in 16.67% of cases [117–120]. The first case of EMPD treated with a complete hormonal blockade was a male patient who received chlormadinone acetate daily and leuprorelin acetate subcutaneous monthly, presenting a paradoxical response, with progression on skin lesions and PR in lymph nodes [121]. In another case, a man with a scrotal mEMPD, which expressed estrogen receptor alfa, and a metastatic prostate cancer, was treated with tamoxifen and bicalutamide concomitantly, with PR for 6 months [122].

Another male patient with mEMPD with overexpression of estrogen and progesterone receptors (30.8% and 1.1% of tumor cells with an Allred score of 5/8 and 4/8 respectively) was treated with first-line tamoxifen. It resulted in a PR which endures after 23 months of ongoing treatment [123].

Inhibition of the PI3K/AKT/mTOR pathway has also been explored in a case reported by Yin et al. The patient presented a HER2 negative EMPD with neuroendocrine features and upregulated PI3K/AKT/mTOR pathway due to a mutation in AMER1 (an inhibitor of PI3K phosphorylation). The combination of anlotinib (a multikinase inhibitor) and tislelizumab (an antibody which minimizes the binding to FcγR on macrophages in order to limit antibody-dependent phagocytosis) rendered the patient a significantly improved DFS of 8 months in an investigational context [124].

### Other Therapies

Systemic immunotherapy has not been specifically studied in EMPD. Moreover, classic biomarker predictors of response with immune checkpoint inhibitors (ICIs) are usually lacking in EMPD [125, 126]. Low rates of PD-L1 expression have also been reported [127]. Nevertheless, PD-L1 has demonstrated to be a good predictor in very few settings, while in most cases, it fails to accurately anticipate a tumor response. A case of a patient with mEMPD who was treated with ipilimumab and nivolumab was reported in 2021. The patients achieved a PR which endured 5 months even though the treatment had to be stopped due to immunotherapy-related hepatitis [128].

Treatment with ICIs must be evaluated in mEMPD to a greater extent and more studies are warranted. An ongoing study (NCT02834013) is exploring the use of nivolumab plus ipilimumab in rare tumors, including EMPD.

There are other rare cases, with reported responses to agents such as apatinib [129].

### Serum Biomarkers

CEA is a biomarker that is usually elevated in patients with mEMPD, while in those with localized disease typically remains in normal range. It can be used to evaluate the response to treatments in metastatic setting [130]. Cytokeratin 19 fragment 21-1 (CYFRA 21-1) has been reported as a sensitive marker for EMPD that may surpass CEA [131, 132].

## Conclusions

We present an overview on the diagnosis and management of EMPD, both localized and metastatic. Given the low incidence of this disease, there are no randomized clinical trials and virtually all knowledge comes from the publication of retrospective cases and reports. However, some general recommendation can be given for the management of patients with EMPD.

The treatment of choice for localized disease endures to be surgery, where Mohs technique may be preferred in order to reduce recurrences.

Discarding the regional lymph node involvement, especially in cases of invasive EMPD or with foci of adenocarcinoma, is key to reduce distance recurrences. In this sense, PET and ultrasound can be useful tools to detect the possible presence of affected regional nodes. Since the performance of a lymphadenectomy has not demonstrated to improve patient survival, performing a SLNB to decide on a possible lymphadenectomy is something that must be individualized.

In those cases, in which surgery is not possible, imiquimod, photodynamic therapy, or radiotherapy should be considered alternative. Radiotherapy could be also used as an adjuvant treatment in case of margins affected, locoregional lymph node involvement, multifocal disease, or presence of adenocarcinoma.

In regard to the treatment of metastatic disease, multiple QT regimens have been used, none clearly superior to others. However, the most commonly used drugs have been taxanes, platins, and fluoropyrimidines, either as monotherapy or in various combinations. Anthracyclines may also be active. In a same way to chemotherapy treatment, there are only few reported cases treated with anti-HER2 treatment and androgen and/or estrogen blockade. However, long-lasting responses have been observed with these treatments. This fact, together with the scarcity of therapeutic options, makes us recommend that the overexpression of HER2, androgen, and estrogen receptors should be performed. The use of ICIs should be carefully assessed given the exceptional number of cases existing.

Finally, the use of some biomarker, such as CEA and CYFRA 21-1, could be used to monitoring the response to treatment.

## Case Report

We present the case of a 75-year-old woman with no personal history of interest, who was being monitored for Paget’s disease of the vulva. The patient consulted in December 2017 for worsening of pruritus in the vulvo-vaginal area. A physical examination showed a vulvar neoformation in vaginal introit, with growth into the vagina and superficial extension to the reset of the vulva in the form of Paget’s disease.

A thoraco-abdominal-pelvic CT scan is performed in January 2018, describing pathological-looking adenopathies in the right external iliac-femoral chain of at least 5.5 × 2.6 cm, as well as suspicious ipsilateral inguinal adenopathies of 2.9 × 1.6 cm. There was also a nodular thickening in the left vaginal dome and a slight skin thickening in the vulvar region.

A biopsy of the vaginal introit is performed, showing an infiltration by a carcinoma compatible with primary vulvar. A biopsy of the inguinal adenopathies is also performed, showing large cell infiltrates with vesicular nuclei and macronucleoli forming solid nests, morphology corresponding to a poorly differentiated adenocarcinoma. The immunohistochemical profile (positivity for CK7, EMA, androgen receptors, and CEA; negativity for CK20, estrogen and progesterone receptors, and GCDFP15) orientates to adenocarcinoma metastases of vulvar origin.

**Fig. 2:**
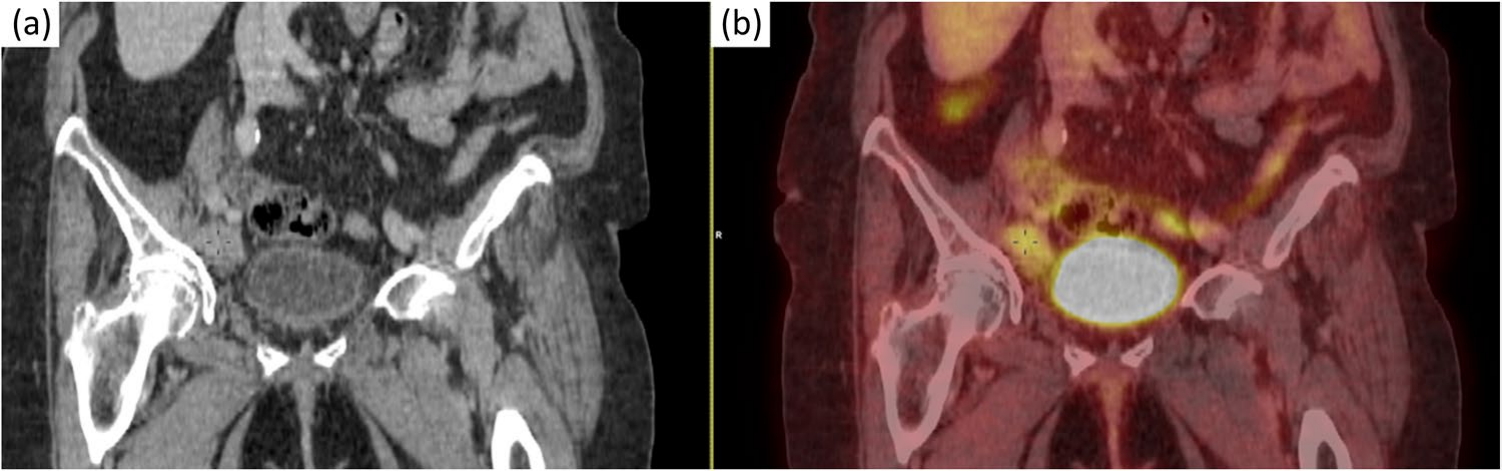
**a** TAC image with nodal affection. **b** PET image with nodal affection

Given these findings, a PET-CT is performed in February 2018, which shows uptake in the following: right hemivulva (SUVmax 3.8), left vaginal dome (2.8 × 2.1 cm, SUVmax 6.6), right inguinal adenopathies (2.7 × 1.7 cm, SUVmax 2.6), external iliac chain adenopathies (6.2 × 3.1 cm, SUV max 3.4), right common iliac bifurcation adenopathies (1.9 × 1.2 cm, SUV max 2.6) (Fig. 2).

With the diagnosis of vulvar mEMPD, due to lymph node involvement, it was decided to start a first-line treatment of chemotherapy with carboplatin AUC-4 plus paclitaxel 135 mg/m^2^ every 3 weeks. The doses were reduced with respect to the classic schemes of ovarian cancer due to the fragility of the patient.

The patient began treatment in March 2018. After 3 cycles, in the reassessment CT scan, a PR at the lymph node level was observed and it was maintained during 3 more cycles.

It was decided, in a multidisciplinary session, to administer consolidation radiotherapy. The administered treatment consisted of 50.4 Gy (1.8 Gy/fraction) on the vulvar area and pelvic and inguinal lymph node chains, with concomitant boost up to 61.6 Gy (2.2 Gy/fraction) on macroscopic lymph nodes and implant in vaginal vault. The treatment was administered in 28 sessions. In the first CT scan after radiotherapy, the patient maintained a PR. However, in February 2019, she presents hepatic progression.

**Fig. 3:**
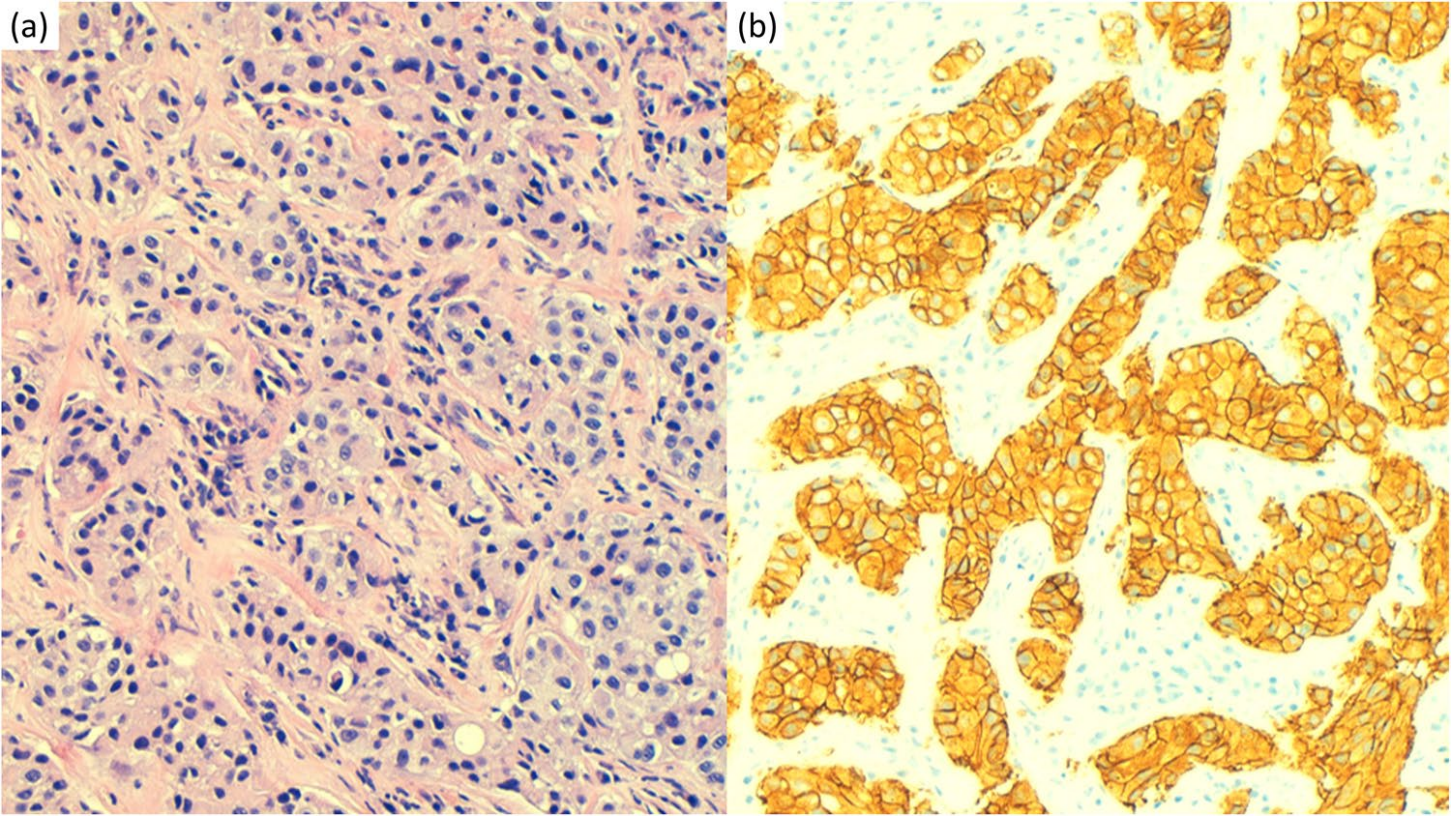
**a** Paget disease (hematoxylin and eosin): nests of tumoral cells with pale cytoplasm infiltrating the vulvar dermis. **b** HER-2 immunohistochemistry (HercepTest, Agilent) scored as positive 3+

At this time, determination of HER2 is requested, resulting positive by immunohistochemistry with a value of 3+ (Fig. 3). It is then decided to initiate a second line of treatment with trastuzumab at a dose of 600 mg subcutaneously every 21 days, and paclitaxel 80 mg/m^2^ on days 1, 8, and 15 every 21 days. Paclitaxel was suspended from the 2nd cycle due to grade 2 neurotoxicity. The patient continued exclusively with trastuzumab 600 mg subcutaneously every 21 days, receiving two more cycles. The patient presents hepatic and nodal progression in July 2019. At this point, the patient moves to another state, with loss to follow-up.
