# Correction to: Extramammary Paget Disease: a Therapeutic Challenge, for a Rare Entity

**DOI:** 10.1007/s11912-023-01456-8

**Published:** 2023-09-11

**Authors:** Jesús Chamorro Pérez, Alfonso Cortes Salgado, Belén Perez‑Mies, Jose Antonio Domínguez Rullán, Odile Ajuria‑Illarramendi, Eva María Guerra Alia, Juan José Serrano Domingo

**Affiliations:** 1 Medical Oncology Department, Ramón y Cajal University Hospital, Carretera de Colmenar Viejo, Km 9.100, 28034 Madrid, CP Spain; 2 Pathology Department, Ramón y Cajal University Hospital, Carretera de Colmenar Viejo, Km 9.100, 28034 Madrid, CP Spain; 3 Radiation Oncology Department, Ramón y Cajal University Hospital, Carretera de Colmenar Viejo, Km 9.100, 28034 Madrid, CP Spain; 4 Nuclear Medicine Department, Ramón y Cajal University Hospital, Carretera de Colmenar Viejo, Km 9.100, 28034 Madrid, CP Spain

**Correction to: Current Oncology Reports**

10.1007/s11912-023-01434-0

The original version of this article contained a mistake in the authors’ names. Many of the authors’ names were reversed (i.e., surname listed before given name).

The authors’ names shown above are correct, and the original article has been corrected.

